# From Theory to Practice: Translating Whole-Genome Sequencing (WGS) into the Clinic

**DOI:** 10.1016/j.tim.2018.08.004

**Published:** 2018-12

**Authors:** Francois Balloux, Ola Brønstad Brynildsrud, Lucy van Dorp, Liam P. Shaw, Hongbin Chen, Kathryn A. Harris, Hui Wang, Vegard Eldholm

**Affiliations:** 1 UCL Genetics Institute, University College London, Gower Street, London WC1E 6BT, UK; 2 Infectious Diseases and Environmental Health, Norwegian Institute of Public Health, Lovisenberggata 8, Oslo 0456, Norway; 3 Department of Clinical Laboratory, Peking University People’s Hospital, Beijing, 100044, China; 4 Great Ormond Street Hospital NHS Foundation Trust, Department of Microbiology, Virology & Infection Prevention & Control, London WC1N 3JH, UK; 5 These authors made equal contributions

## Abstract

Hospitals worldwide are facing an increasing incidence of hard-to-treat infections. Limiting infections and providing patients with optimal drug regimens require timely strain identification as well as virulence and drug-resistance profiling. Additionally, prophylactic interventions based on the identification of environmental sources of recurrent infections (e.g., contaminated sinks) and reconstruction of transmission chains (i.e., who infected whom) could help to reduce the incidence of nosocomial infections. WGS could hold the key to solving these issues. However, uptake in the clinic has been slow. Some major scientific and logistical challenges need to be solved before WGS fulfils its potential in clinical microbial diagnostics. In this review we identify major bottlenecks that need to be resolved for WGS to routinely inform clinical intervention and discuss possible solutions.

## Highlights

In principle, WGS can provide highly relevant information for clinical microbiology in near-real-time, from phenotype testing to tracking outbreaks.

However, despite this promise, the uptake of WGS in the clinic has been limited to date, and future implementation is likely to be a slow process.

The increasing information provided by WGS can cause conflict with traditional microbiological concepts and typing schemes.

Decreasing raw sequencing costs have not translated into decreasing total costs for bacterial genomes, which have stabilised.

Existing research pipelines are not suitable for the clinic, and bespoke clinical pipelines should be developed.

## The Lure of WGS in Clinical Microbiology

Thanks to progress in high-throughput sequencing technologies over the last two decades, generating microbial genomes is now considered neither particularly challenging nor expensive. As a result, **whole-genome sequencing (WGS)** (see Glossary) has been championed as the obvious and inevitable future of diagnostics in multiple reviews and opinion pieces dating back to 2010 1, 2, 3, 4. Despite enthusiasm in the community, WGS diagnostics has not yet been widely adopted in clinical microbiology, which may seem at odds with the current suite of applications for which WGS has huge potential, and which are already widely used in the academic literature. Common applications of WGS in diagnostic microbiology include isolate characterization, **antimicrobial resistance (AMR)** profiling, and establishing the sources of recurrent infections and between-patient transmissions. All of these have obvious clinical relevance and provide case studies where WGS could, in principle, provide additional information and even replace the knowledge obtained through standard clinical microbiology techniques. This review reiterates the potential of WGS for clinical microbiology, but also its current limitations, and suggests possible solutions to some of the main bottlenecks to routine implementation. In particular, we argue that applying existing WGS pipelines developed for fundamental research is unlikely to produce the fast and robust tools required, and that new dedicated approaches are needed for WGS in the clinic.

## Strain Identification through WGS

At the most basic level, WGS can be used to characterize a clinical isolate, informing on the likely species and/or subtype and allowing phylogenetic placement of a given sequence relative to an existing set of isolates. WGS-based strain identification gives a far superior resolution compared to genetic marker-based approaches such as **multilocus sequence typing (MLST)** and can be used when standard techniques such as pulsed-field gel electrophoresis (PFGE), variable-number tandem repeat (VNTR) profiling, and MALDI-TOF are unable to accurately distinguish lineages [5]. WGS-informed strain identification could be of particular significance for bacteria with large accessory genomes, which encompass many of the clinically most problematic bacteria, where much of the relevant genetic diversity is driven by differences in the **accessory genome** on the chromosome and/or plasmid carriage.

**Figure 1:**
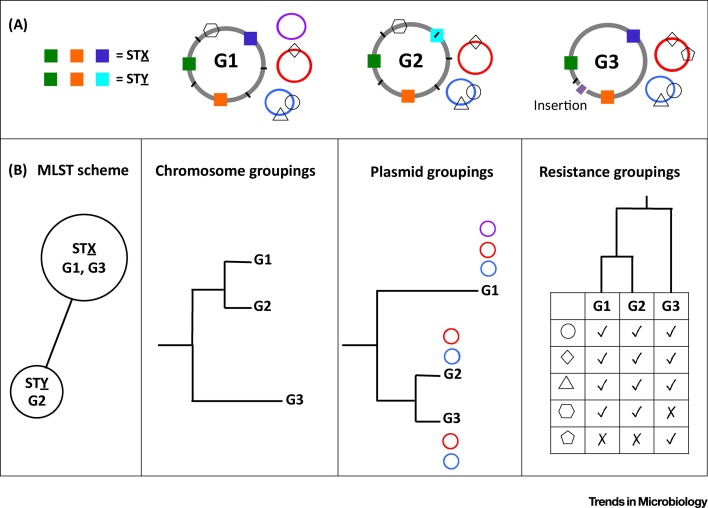
The Challenge of Classifying Organisms with Open Genomes. A hypothetical example of three closely related isolates (G1–G3) collected from the same hospital outbreak. (A) A simplified representation of their genetic makeup. The strains share most of their chromosome, but with G2 having acquired one point mutation (small black line) in one of the genes of the multilocus sequence typing (MLST) typing schemes, and thus being assigned to a different sequence type (ST); G3 also acquired an insertion on the chromosome. Point mutations on the chromosome are represented by short black lines. Additionally, all three strains share two plasmids (red and blue) carrying antimicrobial resistance (AMR) elements (shapes), and G1 has an additional private plasmid (purple). (B) The schematic grouping of these three strains based on MLST typing, chromosomal genetic distances, plasmid similarity, and AMR profile.

Somewhat ironically, the extremely rich information of WGS data, with every genome being unique, generates problems of its own. Clinical microbiology tends to rely on often largely *ad hoc* taxonomical nomenclature, such as biochemical serovars for *Salmonella enterica* or mycobacterial interspersed repetitive units (MIRUs) for *Mycobacterium tuberculosis*. While the rich information contained in WGS should in principle allow superseding traditional taxonomic classifications 6, 7, defining an intuitive, meaningful and rigorous classification for genome sequences represents a major challenge. For strictly clonal species, which undergo no **horizontal gene transfer (HGT)**, such as *M. tuberculosis*, it is possible to devise a ‘natural’ robust phylogenetically based classification [8]. Unfortunately, organisms undergoing regular HGT, and with a significant accessory genome, do not fall neatly into existing classification schemes. In fact, it is even questionable whether a completely satisfactory classification scheme could be devised for such organisms, as classifications based on the core genome, accessory genome, housekeeping genes (MLST), genotypic markers, plasmid sequence, **virulence** factors or AMR profile may all produce incompatible categories (Figure 1).

## Predicting Phenotypes from WGS

Beyond species identification and characterization, genome sequences provide a rich resource that can be exploited to predict the pathogen’s phenotype. The main microbial traits of clinical relevance are AMR and virulence, but may also include other traits such as the ability to form biofilms or survival in the environment. Sequence-based drug profiling is one of the pillars of HIV treatment and has to be credited for the remarkable success of antiretroviral therapy (ART) regimes. Prediction of AMR from sequence data has also received considerable attention for bacterial pathogens but has not led to comparable success at this stage.

Resistance against single drugs can be relatively straightforward to predict in some instances. For example, the presence of the *SCCmec* cassette is a reliable predictor for broad-spectrum beta-lactam resistance in *Staphylococcus aureus,* with strains carrying this element referred to as methicillin-resistant *S. aureus* (MRSA). In principle, WGS offers the possibility to predict the full resistance profile to multiple drugs (the ‘resistome’). Possibly the first real attempt to predict the resistome from WGS data was a study by Holden *et al*. in 2013, showing that, for a large dataset of *S. aureus* ST22 isolates, 98.8% of all phenotypic resistances could be explained by at least one previously documented AMR element or mutation in the sequence data [9].

Since then, several tools have been developed for the prediction of resistance profiles from WGS. These include those designed for prediction of resistance phenotype from acquired AMR genes, including ResFinder [10] and ABRicate (https://github.com/tseemann/abricate), together with those also taking into account point mutations in chromosome-borne genes such as Arg-Annot [11], the Sequence Search Tool for Antimicrobial Resistance (SSTAR) [12], and the Comprehensive Antibiotic Resistance Database (CARD) [12]. Of these, ResFinder and CARD can be implemented as online methods that, dependent on user traffic, can be considerably slower than most other tools that only use the command-line. They are, however, superior in terms of broad usability and are more intuitive than, for example, the graphical user interface of SSTAR. Other tools exist for richer species-specific characterization such as PhyResSE [13] and PATRIC-RAST [14]. Further tools have been developed to predict phenotype directly from unassembled sequencing reads, bypassing genome assembly 15, 16.

It has been proposed that WGS-based phenotyping might, in some instances, be equally, if not more, accurate than traditional phenotyping 16, 17, 18, 19. However, it is probably no coincidence that the most successful applications to date have primarily been on *M. tuberculosis* and *S. aureus*, which are characterised by essentially no, or very limited, accessory genomes, respectively. Other successful examples include streptococcal pathogens, where WGS-based predictions and measured phenotypic resistance show good agreement even in large and diverse samples of isolates 20, 21. On the whole, however, predicting comprehensive AMR profiles in organisms with open genomes, such as *Escherichia coli*, where only 6% of genes are found in every single strain [22], is challenging and requires extremely extensive and well curated reference databases.

The transition to WGS might appear relatively straightforward if viewed as merely replacing PCR panels which are already used when traditional phenotyping can be cumbersome and unreliable. However, to put the problem in context, there are over 2000 described β-lactamase gene sequences responsible for multiresistance to β-lactam antibiotics such as penicillins, cephalosporins, and carbapenems [23]. Whilst β-lactam resistance in some pathogens, including *S. pneumoniae*, can be predicted through, for example, penicillin-binding protein (PBP) typing and machine-learning-based approaches [24], the general problem of reliably assigning resistance phenotype based on many described gene sequences is commonplace.

At this stage, many of the AMR reference databases are not well integrated or curated and have no minimum clinical standard. They often have varying predictive ranges and biases and produce fairly inaccessible output files with little guidance on how to interpret or utilise this information for clinical intervention. Perhaps because of these limitations, although of obvious benefit as part of a diagnostics platform, both awareness and uptake in the clinic has been limited.

Additionally, with some notable exceptions, such as the pneumococci [24], most AMR profile predictions from WGS data are qualitative, simply predicting whether an isolate is expected to be resistant or susceptible against a compound despite AMR generally being a continuous and often complex trait. The level of resistance of a strain to a drug can be affected by multiple epistatic AMR elements or mutations [25], the copy number variation of these elements [26], the function of the genetic background of the strain 27, 28, 29, and modulating effects by the environment [30]. The level of resistance is generally well captured by the semiquantitative phenotypic measurement minimum inhibitory concentration (MIC), even if clinicians often use a discrete interpretation of MICs into resistant/susceptible based on fairly arbitrary cut-off values. Quantitative resistance predictions are not just of academic interest. In the clinic, low-level resistance strains can still be treated with a given antibiotic but the standard dose should be increased, which can be the best option at hand, especially for drugs with low toxicity.

The majority of efforts to predict phenotypes from bacterial genomes have been on AMR profiling. Yet, some tools have also been developed for multispecies virulence profiling: the Virulence Factors Database (VFDB) [31] or VirulenceFinder [32] as well as the bespoke virulence prediction tool for *Klebsiella pneumoniae*, Kleborate [33]. One major challenge is that virulence is often a context-dependent trait. For example, in *K. pneumoniae* various imperfect proxies for virulence are used. These include capsule type, hypermucovisity, biofilm and siderophore production, or survival in serum-killing assays. While all of these traits are quantifiable and reproducible, and could thus in principle be predicted using WGS, it remains unclear how well they correlate with virulence in the patient. Given that virulence is one of the most commonly studied phenotypes, yet lacks a clear definition, the general problem of predicting bacterial phenotype from genotype may be substantially more complex than the special case of AMR, which is itself far from solved for all clinically relevant species.

## Tracking Outbreaks and Identifying Sources of Recurrent Infections

Beyond phenotype prediction for individual isolates, WGS has allowed reconstructing outbreaks within hospitals and the community across a diversity of taxa ranging from carbapenem-resistant *K. pneumoniae* 34, 35, 36 and *Acinetobacter baumannii* [37] to MRSA 38, 39, streptococcal disease [40], and *Neisseria gonorrhoea* [41], amongst others. WGS can reveal which isolates are part of an outbreak lineage and, by integrating epidemiological data with phylogenetic information, detect direct probable transmission events 42, 43, 44, 45. Timed phylogenies, for example generated through BEAST 46, 47, can provide likely time-windows on inferred transmissions, as well as dating when an outbreak lineage may have started to expand. Approaches based on **transmission chains** can also be used to identify sources of recurrent infections (so called ‘super-spreaders’), and do not necessarily rely on all isolates within the outbreak having been sequenced, allowing for partial sampling and analyses of ongoing outbreaks [48]. In this way WGS-based inference can elucidate patterns of infection which are impossible to recapitulate from standard sequence typing alone [35].

However, WGS-informed outbreak tracking is usually performed only retrospectively. Typically, the publication dates of academic literature relating to outbreak reconstruction lag greatly, often in the order of at least 5 years since the initial identification of an outbreak 49, 50. Even analyses published more rapidly are generally still too slow to inform on real-time interventions [38]. Some attempts have been made to show that near-real-time hospital outbreak reconstruction is feasible retrospectively 51, 52 or have performed analyses for ongoing outbreaks in close to real-time 53, 54, but these studies are still in a minority and remain largely within the academic literature.

Some of this time-lag probably relates to the difficulty of transmission-chain reconstruction at actionable time-scales. This can be relatively straightforward for viruses with high mutation rates, small genomes, and fast and constant transmission times, such as Ebola [55] and Zika virus [56], but conversely, reconstructing outbreaks for bacteria and fungi poses a series of challenges. Available tools tend to be sophisticated and complex to implement, and the sequence data needs extremely careful quality control and curation. Unfortunately, in some cases insufficient genetic variation will have accumulated over the course of an outbreak, and a transmission chain simply cannot be inferred without this signal 57, 58. Furthermore, extensive within-host genetic diversity (typical in chronic infections) can render the inference of transmission chains intractable [59]. These complexities mean that a ‘one-size fits all’ bioinformatics approach to outbreak analyses simply does not exist.

## The Bonus of Improved Surveillance

One of the key promises of WGS is in molecular surveillance and real-time tracking of infectious disease. This relies on transparent and standardized data sharing of the millions of genomes sequenced each year, together with accompanying metadata on isolation host, date of sampling, and geographic location. With enough data, surveillance initiatives have the potential to identify the likely geographic origin of emerging pathogens and AMR genes, group seemingly unrelated cases into outbreaks, and clearly identify when sequences are divergent from other circulating strains. In a hospital setting, surveillance can help to detect transmission within the hospital and inflow from the community, optimize antimicrobial stewardship, and inform treatment decisions; at national and global scales, it can highlight worldwide emerging trends for which collated evidence can direct both retrospective but also anticipatory policy decisions.

Amongst the most successful global surveillance initiatives and analytical frameworks are those relating specifically to the spread of viruses. Influenza surveillance is arguably the most developed, with large sequencing repositories such as the GISAID database (gisaid.org) and online data exploration and phylodynamics available through web tools such as NextFlu [60] and NextStrain (http://nextstrain.org), which also allows examination of other significant viruses including Zika, Ebola, and avian influenza. Another popular tool for the sharing of data and visualization of **phylogenetic trees** and their accompanying meta-data is Microreact (microreact.org) [61], which also allows for interactive data querying and includes bacteria and fungi. A further tool, predominately for bacterial data, is WGSA (www.wgsa.net). WGSA allows the upload of genome assemblies through a drag-and-drop web browser, allowing for a quick characterization of species, MLST type, resistance profile, and phylogenetic placement in the context of the existing species database based on core genes. At the time of writing WGSA comprises 20 649 genomes predominantly from *S. aureus*, *N. gonorrhoeae*, and *Salmonella enterica* serovar Typhi, together with Ebola and Zika viruses, all with some associated metadata.

Although an exciting initiative, WGSA and associated platforms are still a reasonably long way off characterizing all clinically relevant isolates and often rely entirely on the sequences uploaded already being assembled. More generally, the success of any WGS surveillance is dependent on the timely and open sharing of information from around the globe. While sequence data from academic publications is near systematically deposited on public sequence databases (at least upon publication), such data are near useless if the accompanying metadata (see above) are not also released, as remains the case far too often. Additionally, as more genomes are routinely sequenced in clinical settings as part of standard procedures, ensuring that the culture of sharing sequence data persists beyond academic research will become increasingly important.

## Cost of WGS in the Clinic

**Figure 2:**
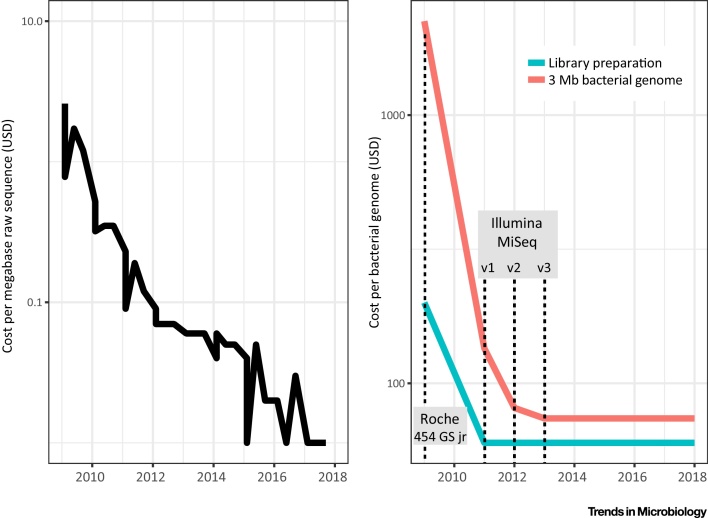
Raw Sequencing Costs Have Dropped over Time but the True Sequencing Cost Per Bacterial Genome Has Stabilised. (A) Sequencing cost per raw megabase (Mb) of DNA sequence between 2009 and 2018. Data from https://www.genome.gov/27541954/dna-sequencing-costs-data/. (B) The evolution of costs for a bacterial genome of 3 Mb sequenced to 50× depth (Illumina) or 30× depth (Roche 454) between 2009 and 2018. The fraction of the total cost (red line) made up of library preparation consumables (blue line) indicates that the drop in raw sequencing costs has had a limited impact on true sequencing costs since 2011, and none after 2013. The cost is based on our calculations for the output and consumable costs for the 454 GS Jr and Illumina Miseq 2 × 150, Miseq 2 × 250 and Miseq 2 × 300, the leading microbiology-scale platforms in terms of output/cost ratio in 2009, 2011, 2015, and 2018 respectively. USD, US$.

For WGS to be routinely adopted in clinical microbiology, it needs to be cost-effective. It is commonly accepted that sequencing costs are plummeting with the National Human Genome Research Institute (NHGRI) estimating the cost per raw megabase (Mb) of DNA sequence to 0.12 USD (www.genome.gov/sequencingcostsdata). This has led to claims that a draft bacterial genome can currently cost less than 1 USD to generate [62]. This is a misunderstanding as one cannot simply extrapolate the cost of a bacterial genome by multiplying a high-throughput per DNA megabase (Mb) sequencing cost by the size of its genome. For microbial sequencing, multiple samples must be multiplexed for cost efficiency, which is easier to achieve in large reference laboratories with high sample turnover. Excluding indirect costs such as salaries for personnel, preparation of sequencing libraries now makes up the major fraction of microbial sequencing costs (Figure 2).

The precipitous drop in the cost of producing raw DNA sequences in recent years (Figure 2A) mostly reflects a massive increase in output with new iterations of Illumina production machines. These numbers ignore all other costs and simply reflect output relative to the cost of the sequencing kits/cartridges. Realistic cost estimates for a microbial genome including library preparation on the best available platforms give a different picture (Figure 2B). Since the introduction of the Illumina MiSeq platform in 2011, new sequencing kits generating higher output have only marginally affected true microbial genome sequencing costs, as library preparation makes up a significant portion of the total (60 USD of a total of 74 USD for a typical bacterial genome in 2018). These costs have remained stable over time and are unlikely to go down significantly in the near future. Indeed, the market seems to be consolidating in fewer hands (e.g., represented by the procurement of KAPA by Roche in 2015), which economic theory predicts will not favor price decrease.

It is also important to keep in mind that these costs are massive underestimates which do not include indirect costs such as salaries for laboratory personnel and downstream bioinformatics. Such indirect costs are difficult to estimate precisely in an academic setting but are far from trivial. Per-genome sequencing and analysis costs are likely to be even higher in a clinical diagnostics environment due to the need for highly standardised and accredited procedures. However, a micro-costing analysis covering laboratory and personnel costs estimated the cost of clinical WGS to £481 per *M. tuberculosis* isolate versus £518 applying standard methods, representing relatively marginal cost savings but with significant time savings [63]. WGS does indeed represent a potentially cost-effective and highly informative tool for clinical diagnostics, but for microbiology-scale sequencing we seem to be in a post-plummeting-costs age.

## Time Scales of WGS-Based diagnostics

One key feature of useful diagnostics tools is their ability to rapidly inform treatment. Most applications of WGS so far have been for lab-cultured organisms (bacteria and fungi). Traditional culture methods require long turnaround time, with most bacterial cultures taking 1-5 days, fungal cultures 7-30 days, and mycobacterial cultures up to 14-60 days. In this scenario, WGS is used as an adjunct technology primarily to provide information on the presence of AMR and virulence genes, which is particularly useful for mechanisms that are difficult to determine phenotypically (e.g. carbapenem resistance). This use of WGS, whilst solving some of the current clinical problems, does not speed up the diagnosis of infection; it is more the case that new technology is replacing some of the more cumbersome laboratory techniques whilst providing additional information.

WGS is more appealing as a microbiological fast diagnostics solution when combined with procedures that circumvent (or shorten) the traditional culture step. This can be achieved through direct sampling of clinical material (Box 1) or by using a protocol enriching for sequences of specific organism(s). Such enrichment methods, generally based on the capture of known sequences though hybridization, are a particularly tractable approach for viruses due to their small genome size. For example, the VirCap virome capture method targets all known viruses and can even enrich for novel sequences [64]. Similar methods targeting specific organisms have been developed and successfully deployed, representing an attractive option for unculturable organisms 16, 65, 66, 67, 68.

Box 1 **WGS beyond Single Genomes**

WGS in the strict sense usually refers to sequencing the genome of a single organism, and it is common to distinguish between the sample (the material that has actually been taken from the patient) and the isolate (an organism that has been cultured and isolated from that sample). WGS methods traditionally sequence a cultured isolate to reduce contamination from other organisms, or sometimes rely on enrichment strategies targeting sequences from a specific organism 66, 67. However, this represents only a small fraction of the total microbial diversity present in a clinical sample.

In contrast, metagenomic approaches sequence samples in an untargeted way. This approach is particularly relevant for clinical scenarios where the pathogen of interest cannot be predicted and/or is fastidious (i.e., has complex culturing requirements). Example applications of clinical metagenomics include: when the disease causing agent is unexpected 74, 75; investigating the spread of AMR-carrying plasmids across species [35]; and characterizing the natural history of the microbiome [76]. The removal of the culture requirement can drastically decrease turn-around time from sample to data and enable identification of both rare and novel pathogens. Different samples however present different challenges. Easy-to-collect sample sites (e.g., faeces and sputum) typically also have a resident microbiota, so it can be challenging to distinguish the etiological agent of disease from colonizing microbes. Conversely, sites that are usually sterile (e.g., cerebrospinal fluid, pleural fluid) present a much better opportunity for metagenomics to contribute to clinical care.

Metagenomic data are more complex to analyze than single species WGS data and tend to rely on sophisticated computational tools, such as the Desman software allowing inference of strain-level variation in a metagenomic sample [77]. Such approaches can be difficult to implement, are computationally very demanding, and are unlikely to be deployable in clinical microbiology in the near future, although cloud-based platforms may circumvent the need for computational resources in diagnostic laboratories. Furthermore, some faster approaches for rapid strain characterization from raw sequence reads, such as MASH [78] and KmerFinder 10, 79, could find a use in diagnostics microbiology, with the latter having been shown to identify the presence of pathogenic strains even in culture-negative samples [10].

However, the differences between these methods should not obscure their fundamental similarities. Obtaining single-species genomes from culture is one end of a continuum of methods that stretches all the way to full-blown metagenomics of a sample. In principle, all methods produce the same kind of data: strings of bases. Furthermore, in all cases what is clinically relevant represents only a small fraction of these data. Integrating sequencing data from different methods into a single diagnostics pipeline is therefore an attractive prospect to quickly identify the genomic needles in the metagenomic haystack in a species-agnostic manner. For example, the presence of a particular antibiotic-resistance gene in sequencing data may recommend against the use of that antibiotic; whether the gene is present in data from a single-species isolate or from metagenomes is irrelevant. As an example, Leggett *et al*. used MinION metagenomic profiling to identify pathogen-specific AMR genes present in a faecal sample from a critically ill infant all within 5 h of taking the initial sample [80].

Alt-text: Box 1

Relative to the time required for culture and downstream analysis of the data, variation in the speed of different sequencing technologies is relatively modest. There is considerable enthusiasm for the Oxford Nanopore Technology (ONT) which outputs data in real time, although the ONT requires a comparable amount of time to the popular Illumina Miseq sequencer to generate the same volume of sequence data. Sequencing on the MiSeq sequencer takes between 13 to 56 hours, but as run time correlates with sequence output and read length, researchers tend to systematically favour runs of longer duration.

## Ethical Considerations

In the context of this review, genetic material from the human patient present in clinical samples represents contamination, a major obstacle to obtaining a high yield of microbial DNA. Protocols exist to deplete human DNA prior to sequencing 69, 70 but these are not completely problem-free as the depletion protocol is likely to bias estimates of the microbial community, and some human reads will likely remain. In particular, levels of human DNA are significantly higher in faecal samples from hospitalized patients compared to healthy controls [71], suggesting that the problem is exacerbated in clinical settings. Therefore, the ethical and legal issues raised by introducing human WGS into routine healthcare [72] cannot be avoided by microbially focused clinical metagenomics. Dismissing these concerns as minor may be an option for academic researchers uninterested in these human data, but it is naive to think that hospital ethics committees will share this view. Even in the absence of human DNA, metagenomic samples from multiple body sites can be used to identify individuals in datasets of hundreds of people [73]. Managing clinical metagenomics data in light of these concerns should be taken seriously, not only as a barrier to implementation but because of the real risks to patient privacy.

## Bespoke Pipelines for Genomics in Clinical Microbiology

**Figure 3:**
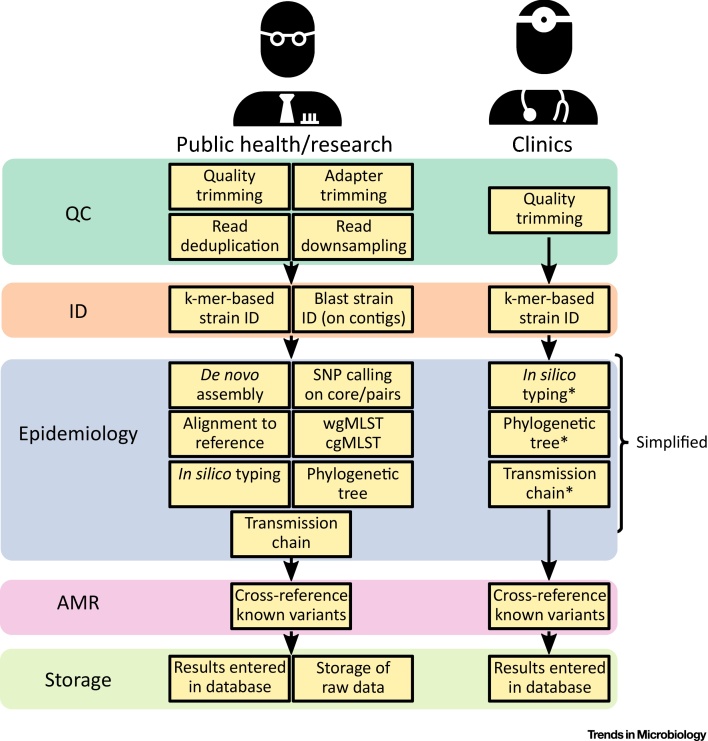
The Standard WGS Research Bioinformatics Pipeline Can Be Modified for Clinical Use. This schematic shows common steps used in public health and/or research together with suggested modifications and omissions for clinical real-time implementation. Steps on the right marked with an asterisk represent simplified versions optimised for speed. cgMLST, core genome multilocus sequence typing; SNP, single-nucleotide polymorphism; wgMLST, whole genome multilocus sequence typing.

A major problem in the analysis of WGS data is that there are currently very few (if any) accepted gold standards. The fundamental steps of WGS analyses in microbial genomics tend to be similar across applications and usually consist of the following steps: sequence data quality control; identification/confirmation of the sequenced biological material; characterization of the sequenced isolate (including typing efforts as well as characterization of virulence factors and putative AMR elements/mutations); epidemiologic analysis; and finally, storage of the results (Figure 3). However, how these analyses are implemented varies widely, both between microbial species and human labs. Despite some commercial attempts at one-stop analysis suites such as Ridom Seqsphere+ (http://www.ridom.com/seqsphere/), most laboratories use a collection of open-source tools to perform particular subanalyses. Typically, these tools are then woven together into a patchwork of software (a ‘pipeline’). The idea of a pipeline is to allow within-laboratory standardized analysis of batches of isolates with relatively little manual bioinformatics work. Such pipelines can be highly customizable for a wide range of questions. There are also some communal efforts at streamlining workflows across laboratories. As an example, Galaxy (https://usegalaxy.org) is a framework that allows nonbioinformaticians to use a wide array of bioinformatics tools through a web interface.

One major limitation to rapidly attaining useful information in a clinical setting is that analysis pipelines for microbial genomics have generally been developed for fundamental research or public health epidemiology [81]. This usually means that the pipeline permits a very thorough and sophisticated workflow with a large number of options and moving parts. For example, at the time of writing (May, 2018), the ‘QC and manipulation’ step in Galaxy alone consists of 35 different tools, tests, and workflows that can be applied to an input sequence. While this is desirable from a researcher’s perspective, it is clearly prohibitive for real-time analysis in a clinical setting. A user requires in-depth knowledge about the purpose each tool serves, the relative strengths and weaknesses of each approach, and a functional understanding of the important parameters. Furthermore, most analysis pipelines require proficiency in Linux systems and navigating the command line, something clinical microbiologists are rarely trained for.

The road to stringent, exhaustive analysis of WGS data is long and paved with good intentions. In order to move towards real-time interpretable results for clinics it will be necessary to take certain shortcuts. The focus should be on rapid, automated analysis and clear, unambiguous results. Some steps in the pipeline can simply be omitted for clinical purposes. As an example, genome assembly might appear to be a bottleneck for real-time WGS diagnostics, but is probably rarely required; sufficient characterization of an isolate can be made by analysis of the **k-mers** in the raw sequence data, which is orders of magnitude faster. Accurate identification of an isolate can be made rapidly with MinHash-based k-mer matching methods such as Mash [78], and AMR elements can be identified from k-mers alone [14]. Another example of a computationally intensive step that could be omitted from a default pipeline is sophisticated phylogenetic inference. Best practice for the creation of phylogenetic trees may involve evaluating the individual likelihood of a very wide range of possible trees given a sequence alignment or other distance metric, repeated for thousands of bootstrapped replicates, giving a tree with high confidence but with extreme computational time costs. A clinical pipeline could use much faster approaches and still provide an informative phylogenetic tree [82].

In Figure 3 we outline our schematic vision of a computational pipeline specific to diagnostics in clinical microbiology. The clinical pipeline would only encompass a small subset of the research pipeline aimed at generating rapid and interpretable output. For epidemiological inference, pairwise distances between strains would be computed as a matrix of Jaccard distances on the shared proportion of k-mers as outputted by Mash [78]. This matrix could be used to generate a phylogenetic tree using a computationally inexpensive method (e.g., neighbor-joining). Additionally, a correlation between pairwise genetic distance and sampling date could be performed to test for evidence of temporal signal in the data (i.e., accumulation of a sufficient number of mutations over the sampling period). In the presence of temporal signal, the user would be provided with a transmission chain based on a fast algorithm such as Seqtrack [83].

Any bespoke pipeline for clinical diagnostics would need to be linked with regularly updated multispecies databases containing information about the latest developments in typing schemes, as well as clinically important factors such as AMR determinants. Results would have to be continuously validated, and international accreditation standards met at regular intervals. At a national level, accreditation bodies (e.g., UKAS in the UK) may lack the expertise required. In our experience, many promising databases have collapsed after funding expired or the responsible postdoc left for another job. If WGS is ever to make it into the clinic it will be necessary to secure indefinite funding of both infrastructure and personnel for such databases.

The lack of uptake of WGS-based diagnostics may also be in part due to an understandable desire to maintain the ‘status quo’ in a busy hospital environment with already established treatment and intervention systems. Additionally, and perhaps significantly, it also highlights the difficulty to communicate the potential benefits of WGS to the day-to-day life of a clinic. The main proponents of WGS tend to be based in the public health/research environment and are rarely actively involved in clinical decision-making. This in itself can present something of a language barrier, challenging meaningful dialogue over how adoption of new approaches can lead to quantifiable improvements in existing systems. Further, the physical planning, implementation and integration of WGS diagnostics may be unlikely to succeed without carefully planned introduction and continued training of its user base. This is of course challenged by the already resource-limited infrastructure of many clinical settings.

## Concluding Remarks

Despite its immense promise and some early successes, it is difficult to predict if and when WGS will completely supersede current standards in clinical microbiology. There are several major bottlenecks to its implementation as a routine approach to diagnose and characterise microbial infections (see Outstanding Questions). These include, among others: the current costs of WGS, which remain far from negligible despite a common belief that sequencing costs have plummeted; a lack of training in, and possible cultural resistance to, bioinformatics among clinical microbiologists; a lack of the necessary computational infrastructure in most hospitals; the inadequacy of existing reference microbial genomics databases necessary for reliable AMR and virulence profiling; and the difficulty of setting up effective, standardized, and accredited bioinformatics protocols.

Focusing in the near future on WGS applications that fulfil unmet diagnostic needs and demonstrate clear benefits to patients and healthcare professionals will help to drive the cultural changes required for the transition to WGS in clinical microbiology. However, irrespective of how this transition occurs and how complete it is, it is likely to feel highly disruptive for many clinical microbiologists. There is also a genuine risk that precious knowledge in basic microbiology will be lost after the transition to WGS, particularly if investment prioritises new technology at the expense of older expertise. More positively, irrespective of the future implementation of WGS in clinical microbiology, we should not forget that the availability of extensive genomic data has been instrumental in the development of a multitude of routine non-WGS typing schemes.

Efforts to develop WGS-based microbial diagnostics have unsurprisingly focused on high-resource settings. Though, we can see an opportunity for low-/medium-income countries to get up to speed with the latest WGS-based developments in real-time clinical diagnostics, rather than adopting classical microbiological phenotyping which might eventually be largely phased out in high-income countries. One precedent for the successful adoption of a technology without transitions through its acknowledged historical predecessors is the widespread use of mobile phones in Africa. This has greatly increased communication and allowed access to e-banking, despite the fact that many people previously had no traditional bank account and only limited access to landlines. Most hospitals in the developing world do not currently benefit from a clinical microbiology laboratory. The installation of a molecular laboratory based around a standard sequencer, such as a benchtop Miseq, might constitute an ideal investment, as it is neither far more expensive nor more complex than setting up a standard clinical microbiology laboratory.

Outstanding Questions Can WGS be used to develop robust classification schemes that account for the genetic diversity of organisms with open genomes?

Which clinically relevant phenotypes can be reliably predicted using WGS, and for which organisms?

How can phylogenetic analyses of outbreaks be speeded up to meaningfully contribute to infection control at actionable time scales?

How can publicly available databases be reliably maintained to the required clinical accreditation standards over long time periods?

Will the true cost of generating a bacterial genome remain stable as the sequencing market consolidates in fewer hands?

How can clinical metagenomic data be managed safely in line with the ethical considerations applicable to identifiable human DNA?

How can unwieldy bioinformatics pipelines developed with academic research in mind be adapted for a clinical setting?

Can current expertise in traditional clinical microbiology be maintained in the transition to WGS?

## Glossary

**Accessory genome**: the variable genome consisting of genes that are present only in some strains of a given species. Many of the organisms representing the most severe AMR threats are characterised by large accessory genomes containing important components of clinically relevant phenotypic diversity.
**Antimicrobial resistance (AMR)**: the ability of a microorganism to reproduce in the presence of a specific antimicrobial compound. Also referred to as antibiotic resistance (ABR or AR). The sum of the detected AMR genes in a sequenced isolate is sometimes referred to as the resistome.
**Horizontal gene tranfer (HGT)**: the transmission of genetic material laterally between organisms outside ‘vertical’ parent-to-offspring inheritance, including across species boundaries. Genetic elements related to clinically relevant phenotypes such as AMR and virulence are often transmitted via HGT.
**K-mer**: a string of length k contained within a larger sequence. For example, the sequence ‘ATTGT’ contains two 4-mers: ‘ATTG’ and ‘TTGT’. The analysis of the k-mer content of raw sequencing reads allows for rapid characterization of the genetic difference between isolates without the need for genome assembly.
**Multilocus sequence typing (MLST)**: a scheme used to assign types to bacteria based on the alleles present at a defined set of chromosome-borne housekeeping genes. Also referred to as sequence typing (ST).
**Phylogenetic tree**: a representation of inferred evolutionary relationships based on the genetic differences between a set of sequences. Also referred to as a phylogeny.
**Transmission chain**: the route of transmission of a pathogen between hosts during an outbreak. This can often be characterized using WGS compared to traditional epidemiological inference based on, for example, tracing contacts between patients.
**Virulence**: broadly, a pathogen's ability to cause damage to its host, for example through invasion, adhesion, immune evasion, and toxin production. However, virulence is currently loosely defined by indirect proxies either phenotypically (e.g., through serum-killing assays) or genetically (e.g., by the presence of genes involved in capsule synthesis or hypermucosvisity).
**Whole-genome sequencing (WGS)**: the process of determining the complete nucleotide sequence of an organism’s genome. This is generally achieved by ‘shotgun’ sequencing of short reads that are either assembled *de novo* or mapped onto a high-quality reference genome.
